# Supplementary material for: Complexome profiling of the Chlamydomonas psb28 mutant reveals TEF5 as an early PSII assembly factor
Source: Plant Cell. 2025 Mar 17;37(6):koaf055. doi: 10.1093/plcell/koaf055 (PMC12164590; doi:10.1093/plcell/koaf055)
Supplement: koaf055_Supplementary_Data [file koaf055_supplementary_data.zip › psb28_interactive.zip!/plots/intensity/qualityPlots/peptides/ORF1995_Cre-1.g000035.t1.p1.html]

### Cre-1.g000035.t1.p1

Trivial name: ORF1995  
SeqCoverage [median]: 19.40  
Mass: 232.19 kDa  
  
 

| Sequence | MissedCleavage | Mass [Da] | wtIntensity | muIntensity | Score |
| --- | --- | --- | --- | --- | --- |
| AAMQTNPAEAR | 0 | 1158.55 | 466.52 | 0.00 | 98.05 |
| DYVEITHK | 0 | 1003.50 | 391.18 | 734.65 | 89.55 |
| EFQQVLKPLQR | 1 | 1384.78 | 543.30 | 4951.67 | 72.01 |
| EQLNQEQTK | 0 | 1116.54 | 410.99 | 0.00 | 71.38 |
| EQLNQEQTKELNK | 1 | 1600.81 | 0.00 | 946.22 | 71.81 |
| FISTSPLASTESKK | 1 | 1494.79 | 857.23 | 1159.62 | 41.09 |
| FLLSEHYDTLR | 0 | 1392.70 | 0.00 | 2787.06 | 67.08 |
| FVVTNR | 0 | 734.41 | 0.00 | 2363.66 | 104.94 |
| GFYYYKPTLR | 1 | 1306.67 | 0.00 | 1463.24 | 45.37 |
| HLFAITPK | 0 | 925.54 | 450.57 | 1167.69 | 88.73 |
| IDNITTLQGAFITK | 0 | 1533.84 | 0.00 | 1172.66 | 64.55 |
| ILVMPSNQQNFNNTLDNTK | 0 | 2190.07 | 0.00 | 2288.38 | 87.49 |
| INVQPVGIMPR | 0 | 1222.69 | 0.00 | 1218.83 | 103.83 |
| KLSQAYSTQQLTK | 1 | 1494.80 | 0.00 | 706.27 | 33.20 |
| KQDNQAYLPK | 1 | 1203.62 | 461.87 | 1455.43 | 70.98 |
| LEIVEMPK | 0 | 957.52 | 633.56 | 0.00 | 86.95 |
| LITINQK | 0 | 828.51 | 1030.51 | 4250.71 | 104.11 |
| LQNTPVQDPNISSPDK | 0 | 1751.87 | 0.00 | 593.37 | 70.49 |
| LQNTPVQDPNISSPDKVLTTAMQK | 1 | 2624.35 | 0.00 | 2418.47 | 98.70 |
| LSQAYSTQQLTK | 0 | 1366.71 | 0.00 | 1537.50 | 63.41 |
| LTVGNQAPYFK | 0 | 1236.65 | 981.74 | 6043.36 | 128.36 |
| LVGLDNLTK | 0 | 971.57 | 650.04 | 3926.35 | 61.96 |
| NVPTGSSAVIDSTVLR | 0 | 1614.86 | 0.00 | 1009.74 | 49.12 |
| NVVESTLTSLNSDGEAATSQPK | 0 | 2247.09 | 0.00 | 1978.07 | 83.06 |
| QGDFYTLK | 0 | 970.48 | 0.00 | 866.51 | 73.25 |
| QILVTPDAK | 0 | 983.57 | 512.80 | 1419.07 | 138.06 |
| QLNNPANPSLETSTK | 0 | 1612.81 | 0.00 | 1069.63 | 86.91 |
| SHFLTKNEER | 1 | 1259.63 | 0.00 | 298.20 | 51.09 |
| SSLYNQTQLLR | 0 | 1321.70 | 0.00 | 1865.56 | 60.52 |
| TIKPGLIYTNSALR | 1 | 1545.89 | 561.72 | 4943.71 | 60.40 |
| VETNQVYLPEGEK | 0 | 1504.74 | 0.00 | 532.71 | 59.43 |
| VVQEYK | 0 | 764.41 | 662.97 | 1488.59 | 117.70 |
| YLSNLQVNNSTLTNK | 0 | 1707.88 | 577.50 | 1865.50 | 122.34 |
| YLTATR | 0 | 723.39 | 795.14 | 1830.40 | 84.02 |
|  |  |  | 9987.6 | 60352.8 |  |
